# Effect of Fibrillated Cellulose on Lime Pastes and Mortars

**DOI:** 10.3390/ma15020459

**Published:** 2022-01-08

**Authors:** Chiara D’Erme, Walter Remo Caseri, Maria Laura Santarelli

**Affiliations:** 1 DICMA—Department of Chemical Engineering Materials and Environment, Sapienza Università di Roma, Via Eudossiana 18, 00184 Rome, Italy; chiara.derme@uniroma1.it; 2 Laboratory for Multifunctional Materials, Department of Materials, ETH Zürich, Vladimir-Prelog-Weg 5, 8049 Zurich, Switzerland

**Keywords:** lime, mortar, fibre reinforced concrete, natural fibres, cellulose

## Abstract

The use of nanocellulose in traditional lime-based mortars is a promising solution for green buildings in the frame of limiting the CO_2_ emissions resulting from Portland Cement production. The influence of the fibrillated cellulose (FC) on lime pastes and lime-based mortars was studied incorporating FC at dosages of 0%, 0.1%, 0.2% and 0.3 wt% by weight of binder. The lime pastes were subjected to thermal and nitrogen gas sorption analyses to understand if FC affects the formation of hydraulic compounds and the mesoporosities volume and distribution. The setting and early hydration of the mortars were studied with isothermal calorimetry. The mechanical performances were investigated with compressive and three-point-bending tests. Furthermore, fragments resulting from the mechanical tests were microscopically studied to understand the reinforcement mechanism of the fibres. It was found that 0.3 wt% of FC enhances the flexural and compressive strengths respectively by 57% and 44% while the crack propagation after the material failure is not affected.

## 1. Introduction

Cement is the most widely used building material and its production is responsible for a large share of greenhouse gas emissions. It has been estimated that cement production can be accounted for about 5% of the total anthropogenic CO_2_ emissions and averagely 12–15% of the industrial energy consumption of each country [1,2,3]. For this reason, several efforts have been directed on reducing the environmental impact of concrete e.g., replacing or blending ordinary Portland cement with alternative binders or using eco-friendly additives.

The use of fibres in mortars and concrete can help to enhance the properties of the final composites. Natural fibres were commonly employed during ancient times to reinforce and to reduce the shrinkage in mortars and concrete; nowadays their application in modern composites is newly evaluated as they represent a renewable, economical and abundant resource [4] and can become an alternative to the use of other synthetic fibres.

Nanocellulose materials can overall enhance the mechanical properties of modern concrete due to their high aspect ratio and high Young’s modulus [5,6,7]. Moreover, due to their high hygroscopicity they can act as an internal curing agent of cement, preventing self-desiccation and promoting hydration as well as autogenous healing phenomena [8,9,10]. Their application as viscosity modifiers in self-compacting concrete (SCC) allows to stabilise the fresh concrete, inhibiting bleeding and segregation phenomena [11]. Cellulose fibres in concrete can also help tuning the composite porosity and changing its hygrothermal behaviour [12,13,14]. On the other hand, some disadvantages must be considered, e.g., the incorporation of this kind of fibres can highly decrease the workability of the fresh concrete, making the casting process more difficult. Moreover, the results in literature are highly scattered [15] as they strongly depend on the nature of the cellulose fibres employed: the source (e.g., plant, waste materials, bacteria), their production process and the strategy of incorporation used.

The use of nanocellulose in traditional lime-based mortars is a promising solution for green buildings in the frame of limiting the CO_2_ emissions resulting from Portland Cement production [16]. Unlike ordinary Portland cement, lime is fired at lower temperatures (below 1000 °C), causing less CO_2_ emissions. Additionally, slaked lime partially re-absorbs the carbon dioxide emitted during the production process as it recarbonates over its in-use phase, thus resulting in lower net carbon emissions [17,18]. Moreover, lime can as well be used for the retrofit of historical buildings [19], since it is well known that cement-based mortars are not chemically and physically compatible with aged and porous materials [20]. In this context, the use of fibres can indeed help increasing the flexural strength, avoiding crack propagation in mortars and plasters, eventually delaying the need for maintenance interventions. However, to our knowledge, no extensive study of the influence of nano- and micro-fibrillated cellulose materials on the hydration and on the mechanical properties of lime-based mortars has been performed so far. 

This study aims at exploring the performance of cellulose nanomaterials in traditional lime-based mortars in order to understand the composites applicability and potential.

## 2. Materials and Methods

### 2.1. Materials

**Figure 1:**
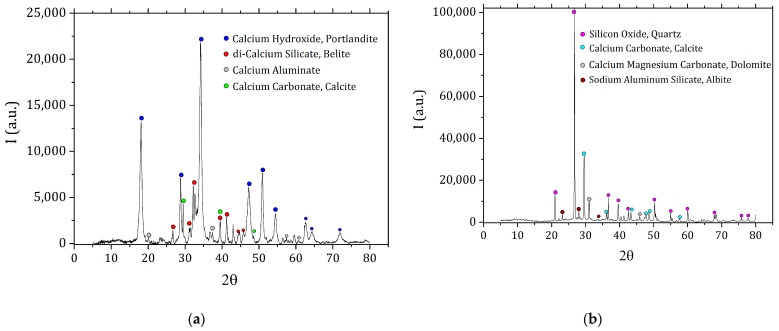
X-ray diffraction patterns on the raw materials: NHL5 (**a**) and quarry sand (**b**).

**Figure 2:**
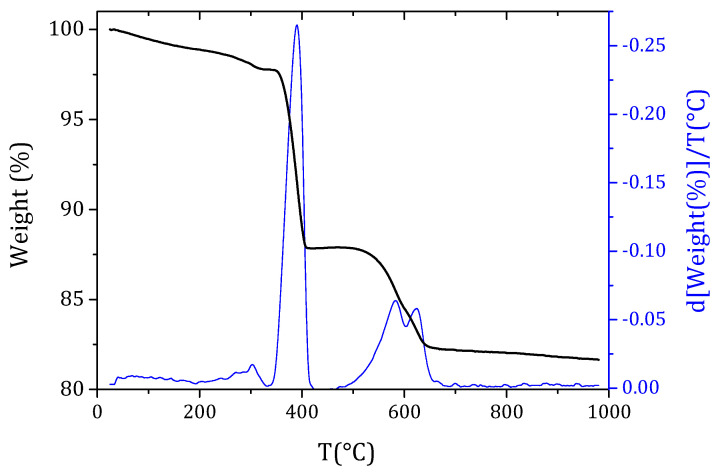
TGA/DTG of NHL5 produced by Otterbein, Germany.

The samples used in the following analysis were prepared with natural hydraulic lime Hydradur NHL 5 (Otterbein, Großenlüder-Müs, Germany) as binder and a 0/4 siliceous quarry sand (Eberhard, Kloten, Switzerland) as aggregate. X-ray diffraction analyses show that the main phases present in NHL5 are portlandite, calcium silicates, calcium aluminates and calcite, while the sand is mainly constituted by quartz and carbonates (calcite and dolomite) and plagioclases (albite) in minor quantities (Figure 1). In thermogravimetric analysis (TGA) of NHL5 the presence of uncalcinated limestone is more evident, the double peak in the derivative (DTG) between 500 °C and 700 °C corresponds to the loss in CO_2_ by decomposition of carbonates (Figure 2).

#### Fibrillated Cellulose

Celova, a type of fibrillated cellulose (FC) produced by Weidmann, (Weidmann, Rapperswil-Jona, Switzerland), was applied for the following experiments. Celova was chosen after preliminary Vicat tests, to evaluate the setting time, and mechanical tests, as described previously [21]. The commercial batch, provided as gel, has an average solid content of 3.3% *w*/*w*. The water retention value (WRV%, in percent) of cellulose was calculated to quantify the maximum amount of water the fibres can retain from a suspension. The as-received gel was diluted to 1% and 2% solid content (*w*/*w*) with distilled water and then sonicated in an ultrasonic bath for 10 min in order to improve the dispersion of the fibres. The suspension was then centrifugated at 4000 rpm (2701 RCF) for 20 min, the supernatant solution disposed and the cellulose pulp dried in an oven at 60 °C until constant mass was reached. The WRV% was calculated according to Equation (1)

(1) $$\mathrm{WRV}\%=\frac{m-m_{dry}}{m_{dry}}\times 100$$

where *m* is the mass of the gel and $m_{dry}$ the mass of the dry sample.

To understand the fibrils morphology, the gel was diluted with deionized water to 0.001% (*w*/*w*) solid content, dropped on a silicon wafer and coated with carbon for scanning electron microscopy (SEM) investigations using a Zeiss Leo-1530 (Zeiss, Oberkochen, Germany).

### 2.2. Methodology and Experiments

#### 2.2.1. Sample Preparation

The lime pastes were prepared by manually mixing the water and the lime, with a ratio of 0.83, in a beaker for 5 min, until a homogeneous paste was obtained. Samples with 0%, 0.1%, 0.2% and a 0.3% of FC, dosed by weight of binder (*w*/*w*), were prepared by pre-dispersing the FC in water using an ultrasonic bath and then incorporated in the lime paste (Table 1). The water content was adjusted subtracting the water introduced with the gel Celova and already absorbed in the fibres. The mixtures were then transferred to a sealed box where a relative humidity (RH) of 65% was maintained with a saturated NaNO_2_ solution and kept at a temperature of 23 ± 1 °C until the designated sample ages for the tests.

**Figure 3:**
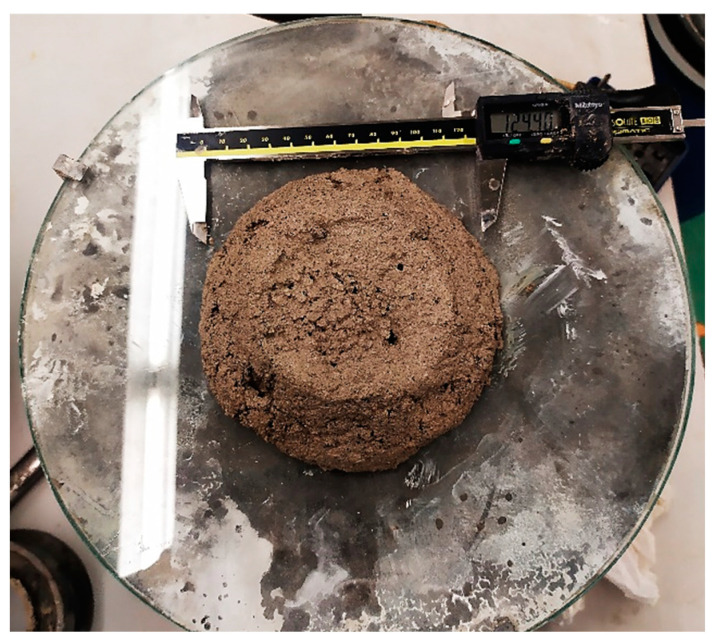
Image of a lime mortar representative slump obtained in agreement with EN 1015-3.

A standard mixer (Hobart N50) was employed to prepare mortars with a binder:aggregate:water volume ratio of 1:1:0.6. Fractions of 0%, 0.1%, 0.2% and a 0.3% of FC dosed by weight of binder (*w*/*w*) were incorporated in the mixtures (Table 2). A polycarboxylate (PCE) superplasticiser, MasterGlenium ACE 30 (Master Builders Solution, Maharashtra, India), dosed by weight of binder (*w*/*w*), was used to increase the mortar workability and to obtain slumps of approximately 125 mm (Figure 3) keeping the same water to binder ratio (w/b). The slumps to evaluate the consistency of the mixes were obtained with the mean of a flow table following the procedure for mortars described in EN 1015-3 [22]. The FC was pre-dispersed in the water and stirred at a speed of 281 rpm for 5 min, before proceeding to the mortar preparation. The process started by mixing the NHL, the water and the superplasticiser for 1 min at low speed (136 rpm). After that, the aggregate was added and blended for 1 min at a speed of 281 rpm. The mixer was stopped for 90 s to scrape the mortar from the walls and finally the mixture was blended at a speed of 281 rpm for 4 min. To prepare the samples for the mechanical tests, fresh mixtures were poured in 20 × 20 × 80 mm moulds. The moulds were filled to the half of their capacity and compacted for 30 s on a vibrating table. Afterwards, a second layer of mixture was poured, and the samples were compacted again. The samples were kept in climatic chamber at 99.9% RH and 20 °C for 5 days before demoulding; after being demoulded they were moved to a climate chamber at 65% RH and 20 °C and cured there until the age for testing was reached.

#### 2.2.2. Hydration Study

**Figure 4:**
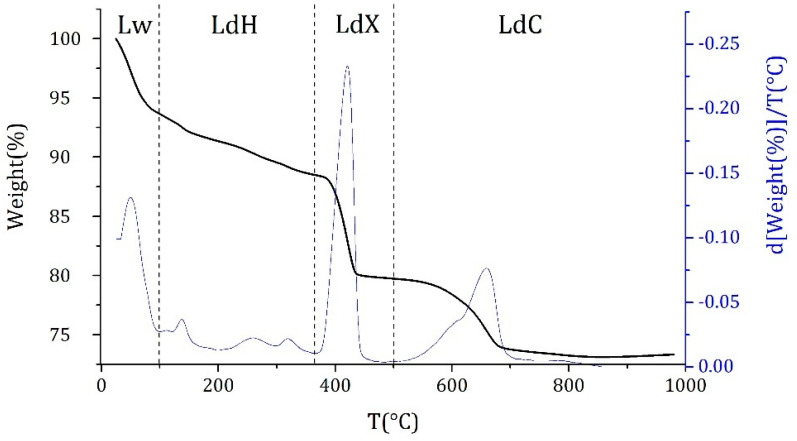
TGA/DTG plot of a lime paste and ranges of temperature related to the principal phenomena occurring in the sample (see text).

Thermogravimetric analyses were performed on the lime pastes to understand if the fibres can affect the hydration mechanisms. At each time a portion of the paste was sampled, crushed and analysed without any further treatment with a Mettler Toledo TGA/DSC 3 + Star^e^ System (Mettler Toledo, Schwerzenbach, Switzerland) under air atmosphere at a heating rate of 10 °C/min, from ambient temperature to 980 °C. It should be noted that in this work no sample processing or drying was made as it has been proved that this can affect the final results and render the hydrates instable [23]. The thermograms were divided in temperature ranges related to four phenomena assumed to occur in the sample (Figure 4) [23,24]. The first peak within the temperature range of 25–105 °C was attributed to free water (Lw) and was not considered in the calculations. The phenomena within the temperature range of 105–370 °C was assumed to represent dehydration reactions (LdH), the third one between 400 °C and 600 °C to the dehydroxylation of portlandite (LdX) and the last region to the decarbonation of calcite and dolomite impurities (LdC). Admittedly this division is somewhat arbitrary since certainly, for instance, the temperature range of free water desorption and dehydration reactions is not sharply separated.

The remaining paste was dried under vacuum (24 mbar) for a minimum of 24 h and then subjected to X-ray diffraction (XRD) measurements performed on a PANalytical Empyrean instrument (Malvern Panalytical, Malvern, UK) equipped with a Cu Kα X-ray tube (45 kV, 40 mA) and a monochromator. 

#### 2.2.3. Nitrogen Gas Sorption

Nitrogen sorption experiments were performed on a Quantachrome Autosorb-iQ-C-XR (Quantachrome Instruments, Graz, Austria) at 77 K. Samples of lime pastes and mortars were crushed into pieces of 12 mm maximum diameter and were later outgassed at 60 °C for a minimum of 24 h, before the gas sorption analyses were performed. The specific surface area of specimens was calculated with the Brunauer-Emmett-Teller (BET) multi-point method. The pore size and pore volume were determined by the density functional theory (DFT) analysis.

#### 2.2.4. Isothermal Calorimetry

Calorimetric measurements were performed on about 70 g of mortar in an isothermal calorimeter TAM Air (Waters GmbH, UB TA Instruments, Eschborn, Germany) at 23 °C. The data were collected up to 30 h as at later stages the heat release was below the machine sensitivity. The calorimetric test was started just at the end of the mixing procedure and after checking the target slump. The exact starting time of the measurement was considered in the calculation. The results were normalised by the dry content of NHL and expressed in terms of heat rate (mW/g) and cumulative heat (J/g).

#### 2.2.5. Mechanical Tests

Mechanical tests were performed using a ZwickRoell universal testing machine with a 20 kN load cell and the results were calculated according to the BS EN 12390 norm [25,26]. Three-point bending tests was carried out for each type of mix at 28 d and at 45 d of curing. The investigation at the longer time of curing was chosen because lime mortars develop their strength more slowly. The support span was set at 60 mm and the load was applied with a rate of 0.5 mm/min. After the flexural test, cubic portions (20 × 20 × 20 mm) of each specimen resulting halves were mechanically tested by compression applying the load at a rate of 1 mm/min.

Furthermore, the curves resulting from the three-point-bending test were analysed to understand if the fibres also affect the brittle behaviour of the material. The experimental displacement data (mm) were transformed in strain ε (%) by the following equation:

(2) $$\varepsilon =\frac{6\Delta l\;d}{l^{2}}\times 100$$

where Δ*l* is the displacement (mm), *d* is the depth or thickness of tested beam (mm) and *l* is the support span (mm). The toughness of the material was estimated by integrating the total area (A) of the stress (MPa)–strain (%) curve, while the area (A_b_) after the break point ε_b_ was calculated to have more information about the fibres possible effect on the crack propagation. The flexural modulus, *E*, was estimated according to Equation (3):

(3) $$E=\frac{1}{4}\frac{l^{3}s}{bd^{3}}\;$$

where *l* is the support span (mm), *b* is the width of test beam (mm), *d* is the depth or thickness of tested beam (mm) and *s* is the slope of the initial straight-line portion of the load (N)—deflection (mm) curve.

The results of the mechanical tests at 45 d were analysed according to Weibull statistics, which is often used to study the failure of brittle materials and that can also describe accurately the failure occurring in concrete [27]. To extrapolate the desired parameters the Weibull function was linearised:

(4) $$ln[ln\left(\frac{1}{1-P_{f}})\right]=m\left(ln\sigma_{f}-ln\sigma_{0}\right)$$

where $P_{f}$ is the probability of failure, calculated according to Equation (5), *m* is the Weibull Modulus (also defined as the shape parameter), $\sigma_{f}$ is the strength at failure, $\sigma_{0}$ is the scale parameter or the characteristic strength for which $P_{f}$ is 63.2%.

(5) $$P_{f}=\left(\frac{i-0.5}{n}\right)$$

where *i* and *n* are respectively the rank and the number of data.

#### 2.2.6. Microscopic Observations

Fragments of mortars obtained from the mechanical test were embedded in epoxy resin to study the morphology of the samples. The samples were optically observed with a Keyence multiscan VHX 6000 microscope (Keyence, Osaka, Japan) and then coated with carbon and subsequently studied by scanning electron microscope (SEM) (Zeiss, Oberkochen, Germany).

The fractured surfaces obtained from the three-point-bending test were analysed using the same Keyence multiscan VHX optical microscope. The instrument can produce a 3D scan image of the sample so that the morphology and the surface roughness can be studied. Eleven samples for the reference and eleven samples with 0.3% FC were optically scanned at 100× magnification to understand if the fibres also affect the crack propagation and therefore the final roughness of the broken samples. The surface roughness parameters calculated with the Keyence built-in software are defined in the following way:

- The arithmetical mean height (*Sa*) is the average value of the absolute value of height at each point, z(x,y), in the defined area (A).
  (6) $$Sa=\frac{1}{A}\iint_{A}^{.}\left|z\left(x,y\right)\right|dxdy$$
- The root mean square height (*Sq*) is the root mean square of height at each point in the area; it is equivalent to the standard deviation of height.
  (7) $$Sq=\sqrt{\frac{1}{A}\iint_{A}^{.}z^{2}\left(x,y\right)dxdy}$$
- The maximum height (*Sz*) is the sum of the maximum peak height (*Sp*) and the maximum valley (*Sv*) depth in the definition area.
  (8) $$Sz=Sp_{}+Sv$$
- The skewness (*Ssk*) is the cubic average of height which is rendered dimensionless with the cube of *S_q_*. It indicates the asymmetric property of height distribution which is centred on the reference surface. When *Ssk* equals 0, it means that the height distribution is symmetric with respect to the reference plane. When *Ssk* < 0, *Ssk* > 0, the height distribution is skewed either higher or lower, relative to the reference plane.
  (9) $$Ssk=\frac{1}{S_{q}^{3}}\left[\frac{1}{A}\iint_{A}^{.}z^{3}\left(x,y\right)dxdy\right]$$
- The kurtosis (*Sku*) is the average fourth power of height which is rendered dimensionless with the fourth power of *S_q_*. It indicates the sharpness of the height distribution. When *Sku* equals 3, it means that the height distribution of a scale-limited surface in characterised by a normal distribution. When *Sku* < 3 or *Sku* > 3, the height distribution takes either a collapsed or sharpened shape.
  (10) $$Sku=\frac{1}{S_{q}^{4}}\left[\frac{1}{A}\iint_{A}^{.}z^{4}\left(x,y\right)dxdy\right]$$

## 3. Results and Discussion

### 3.1. Fibrillated Cellulose

**Figure 5:**
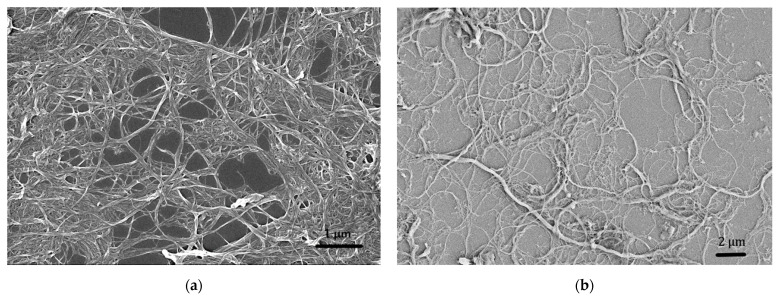
SEM images at different magnifications of a sample of new Celova produced by Weidmann, Switzerland with SE InLens (**a**) and normal SE (**b**) detector; bundles of cellulose fibres above 100 nm arise all over the sample.

The fibres used in this study are composed of a complicated network (Figure 5), as observed in the scanning electron microscope (SEM) images. The primary fibre diameters are below 30 nm, but due to the partial defibrillation bigger bundles of fibres were observed. The aspect ratio is high as the fibre length is in the micrometric scale: however, it was not possible to get an accurate average of the fibre lengths, due to the complicated network they form.

**Figure 6:**
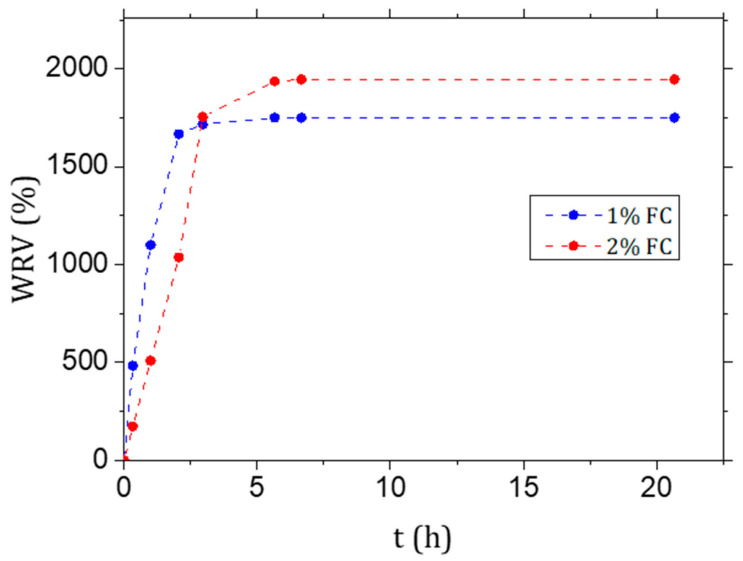
Water retention value (WRV%) of the fibrillated cellulose (FC) gel used to cast the samples diluted to 1% and 2% *w*/*w*.

The water retention value ranges between 1500% and 2000% per mass of dry cellulose (Figure 6). The loss in workability experienced in preparing the mortars and the required increase in the SP content to obtain similar slumps (see Section 2.2.1. Sample Preparation) are thus not only due to the complex 3D net of the FC but also to the high amount of water absorbed by the cellulose. However, since the mechanism of water release is still not clear, the absorbed water was not subtracted from the calculation of the total water to binder ratio.

### 3.2. Hydration Study

Hydraulic lime mainly sets by the reaction of dicalcium silicate (*C*_2_*S*) with water to form calcium silicate hydrates (*CSH*); some hydrated lime (free lime) is also formed in addition to the one already present in the powder before the reaction (Equation (11))

(11) $$C_{2}S+H_{2}O\;\to \;CSH+\;\mathrm{Ca}{\left(\mathrm{OH}\right)}_{2}$$

The lime paste is highly alkaline, but this condition changes progressively on aging because carbon dioxide starts penetrating into the system once the paste is set and transforms the hydrated lime into calcium carbonate. Carbon dioxide also reacts with the hydrated calcium silicate forming calcium carbonate and amorphous silica (*SH*) (Equation (12)).

(12) $$CSH+\mathrm{Ca}{\left(\mathrm{OH}\right)}_{2}+{\mathrm{CO}}_{2}\to {\;\mathrm{CaCO}}_{3}+SH+H_{2}O$$

As the introduced FC has a high WRV%, it was crucial to understand if the setting and hardening reactions in the lime pastes could be affected; more specifically emphasis was put in understanding if the formation of *CSH*, important for the final mechanical strength of the mortar, was inhibited or delayed. It has been discussed that in Portland cement-based mortars and concretes, natural fibres can slow down the hydration at early stages, but natural fibres can also act as an internal reservoir of water avoiding the desiccation of composites and therefore at last promoting the hydration of mortars [9].

**Figure 7:**
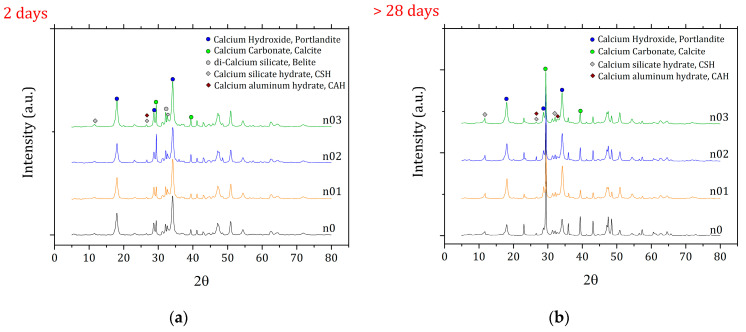
XRD pattern of the four pastes at 2 d (**a**) and after 28 d of curing (**b**).

The XRD pattern (Figure 7) at 2 d and after 28 d show in all four samples a gradual decrease in the Portlandite content, while at the same time the peaks assigned to calcite increase in intensity. The signals associated to the formation of the hydrates (*CSH* and *CAH* in the form of katoite) slowly increase, too. 

To obtain a quantitative impression of the pastes composition, thermogravimetric analyses (TGA) were performed. The TGA curves show that the formation rate of the hydrates is comparable in all the four samples examined. After 21 d of curing the mass loss between 105 °C and 370 °C fluctuates around 4.5% of the total mass up to 45 d. The fractions of portlandite and of calcite, on the other hand, are different in the samples with and without fibres. In samples with fibres the decrease in portlandite and the increase in the calcite content seem to be slower. At 45 d of curing the LdX for n0 is near 4% and between 5.3% and 6.5% for n01, n02 and n03, while LdC is over 22% for n0 and below 18% for n01, n02 and n03. Further studies at different relative humidities should be conducted to understand if FC can play a role in CO_2_ penetration by affecting the paste porosity or if the water stored in the FC can indeed delay the carbonation by avoiding the self-desiccation.

### 3.3. Nitrogen Gas Sorption

**Figure 8:**
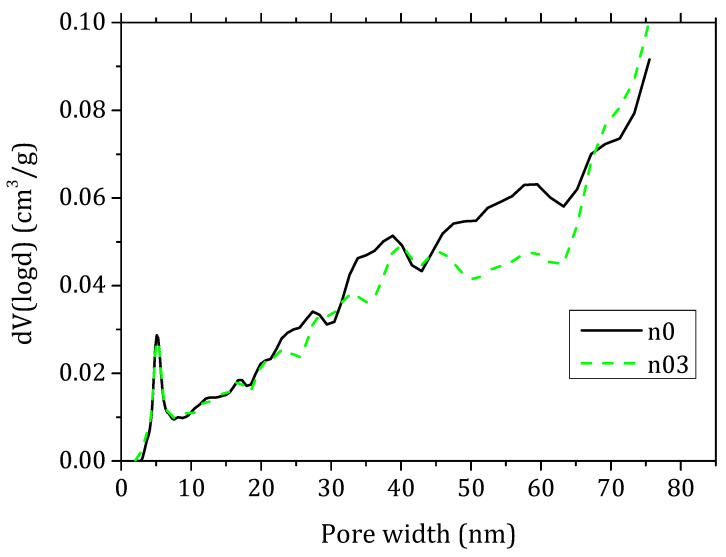
Pore size distribution calculated by DFT method from N_2_ adsorption–desorption isotherms for lime pastes with 0% FC (n0) and 0.3% fc (n03).

The calculated specific surface area for the lime pastes n0, n01, n02 and n03 ranges between 9 m^2^/g and 11 m^2^/g without a significant difference within the accuracy of the measurements. The pores volume distribution is also very similar among the series with the exception of n03 for which the mesoporosities are lower in the range of 20–70 nm (Figure 8). Moreover, the mortars show a similar trend, but the presence of aggregates and inhomogeneities of the samples leads to a higher scatter in the results. These results could indicate that the fibres partially reduce the mesoporosities of the composite.

### 3.4. Isothermal Calorimetry

**Figure 9:**
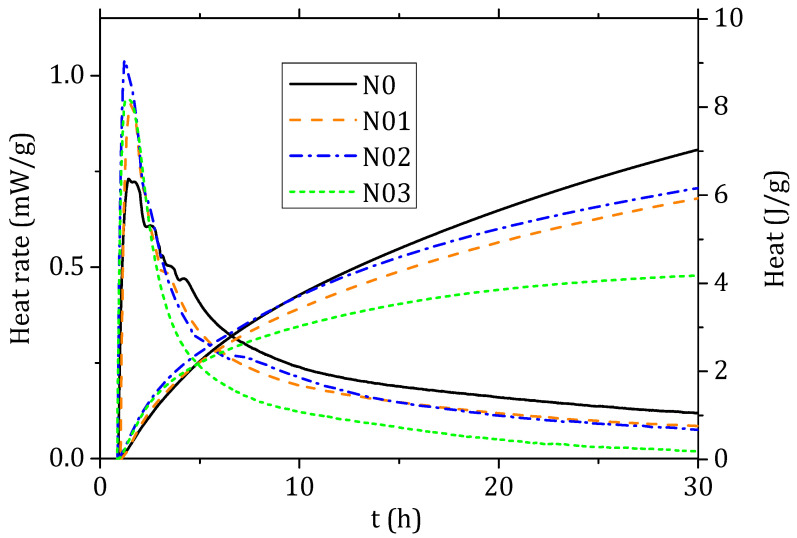
Isothermal calorimetry results for the four different mortar mixtures expressed in heat rate (mW) and heat (J) normalised to the mass of binder.

The specific heat development per gram of binder is presented in Figure 9. A maximum is observed after 1 h. The rapid increase in the heat release can be connected to the heat of wetting [28]. A retardant effect on the setting hydration of cement mortars connected to the use of PCE was often reported [29,30]; this phenomenon was not observed in our lime mixes, despite the difference in the superplasticiser content. On the contrary a slight shift on the left was observed for N02 and N03, the samples with higher superplasticiser content. The maximum heat rate value is higher in samples with FC and could be connected to the partial alkaline hydrolysis of the cellulose by peeling [31]. However, the curve associated to N0 is broader and the cumulative heat recorded is higher.

### 3.5. Mechanical Tests

**Figure 10:**
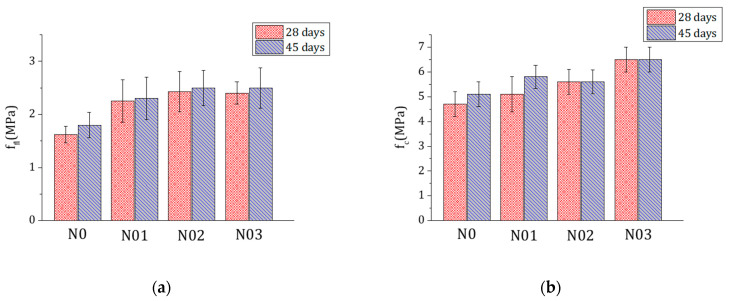
Flexural strength f_fl_ (**a**) and compression strength f_c_ (**b**) of samples with 0% (N0), 0.1% (N01), 0.2% (N02) and 0.3% FC (N03) at 28 d (red) and 45 d (blue).

**Figure 11:**
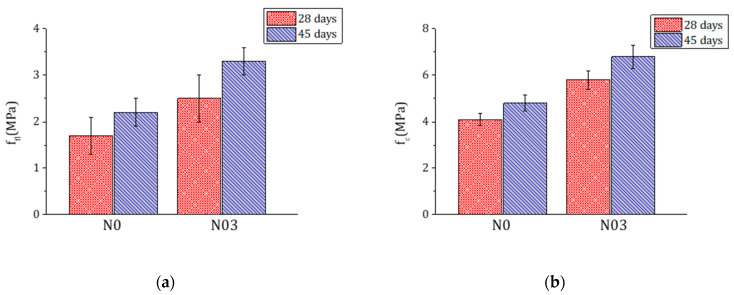
Flexural strength f_fl_ (**a**) and compression strength f_c_ (**b**) of samples with 0% FC (N0) and 0.3% FC (N03) at 28 d (red) and 45 d (blue).

Preliminary tests showed that 0.1% and 0.2% FC affect less significantly the mechanical properties of the mortars, in particular the compression strength (Figure 10). Therefore, the study focused on the comparison between the 0% and 0.3% FC mixtures. Each mix was casted three times to increase the statistics, reduce the dispersion of data (typical of this kind of building materials) and understand the reproducibility of the results. For each mix, 10 and 15 samples were tested respectively at 28 d and at 45 d. The results from the mechanical tests in terms of flexural strength (f_fl_) and compression strength (f_c_) are respectively shown in Table 3 and Table 4. The flexural and compression strength in samples with 0.3% FC are improved respectively by 47% and 41% at 28 d and by 50% and 42% at 45 d (Figure 11). 

**Figure 12:**
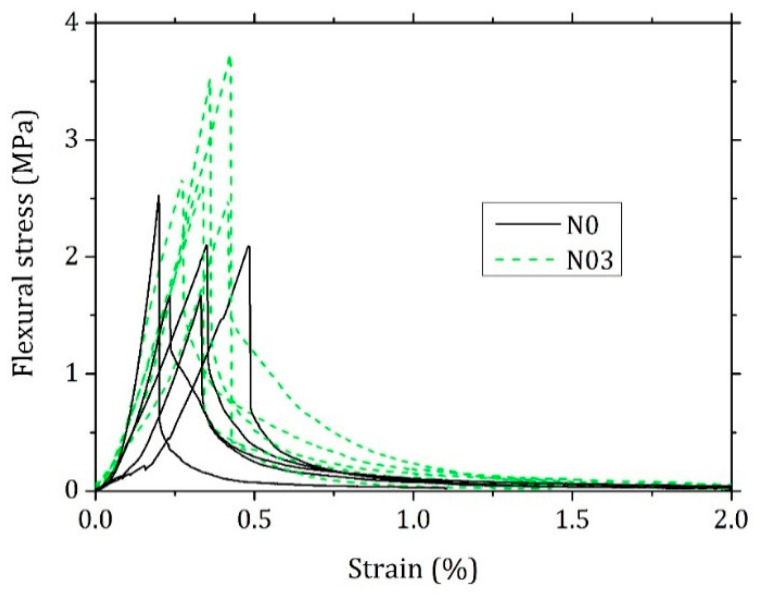
Representative flexural stress–strain curves from the three-point-bending test for samples with 0% (N0) and 0.3% (N03) FC.

Representative experimental flexural stress–strain curves are shown in Figure 12 and were analysed to understand if the fibres also affect the brittle behaviour of the material. The total area under the three-point-bending test curve is also improved in N03 (Table 5). On the other hand, Ab and εb are not significantly affected by the FC, so that the fibres seem to have little effect on the post-crack behaviour and deflection of the composite. The flexural elastic modulus E, on the contrary, is enhanced by 54% indicating the material is behaving in a more rigid way.

The FC effect on the mechanical properties can be connected to the microstructural changes that the fibres can induce in composites and that were already observed in Portland cement concrete. These changes can be due to the higher elastic modulus of the crystalline portion of cellulose nanomaterials (usually in the range of 100–130 GPa) [32], their high specific surface area and the interaction of the surface –OH groups with *CSH* and portlandite that leads to a strong fibre–matrix bonding, which involves a better stress transfer from the matrix to the nanofibers, but in return can also cause an excessive embrittlement of the composite [33,34].

**Figure 13:**
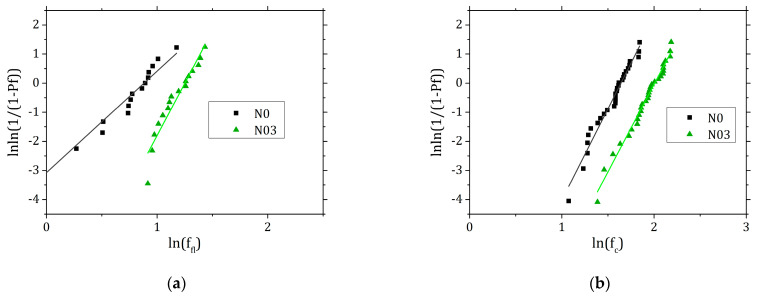
Linear fit of the experimental data from the three-point bending (**a**) and compression (**b**) tests analysed with Weibull statistics.

The Weibull statistics was applied on the data of the mechanical strength at 45 d (Figure 13), a time at which, in the frame of our work, the mortars reached the highest strength. The Weibull analysis confirms the overall enhancement of the mechanical properties (Table 6) determined by the fibres, and the characteristic strength *σ*_0_ is close to the normal distribution averages calculated. The flexural and compressive *σ*_0_ in the N03 series are enhanced respectively by 57% and 44%. The Weibull modulus *m* of the flexural data is considerably higher in samples with 0.3% fibre content, indicating that the distribution is narrower and the results less scattered. A higher Weibull modulus is indeed usually more desirable as the material has a lower probability to fail at a stress much lower than *σ*_0_.

### 3.6. Microscopic Observation

**Figure 14:**
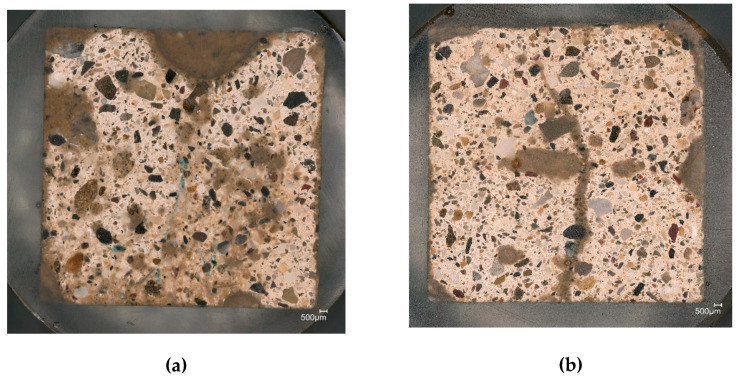
Embedded samples with 0% FC (**a**) and 0.3% FC (**b**) prior to the carbon coating observed with an optical microscope.

Optical microscope observations (Figure 14) showed that both N0 and N03 are homogeneous, and the aggregate is well dispersed in the binder. On the other hand, the samples with FC seem to have less voids and cracks as the embedding resin did not penetrate as much as in N0.

**Figure 15:**
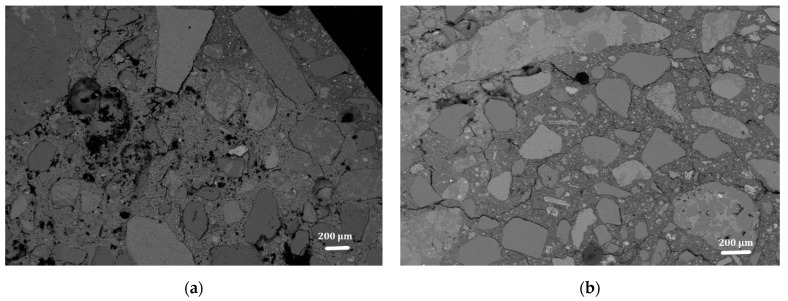
Backscattered electron (BSE) SEM images of mortars with 0% FC (**a**) and 0.3% FC (**b**).

**Figure 16:**
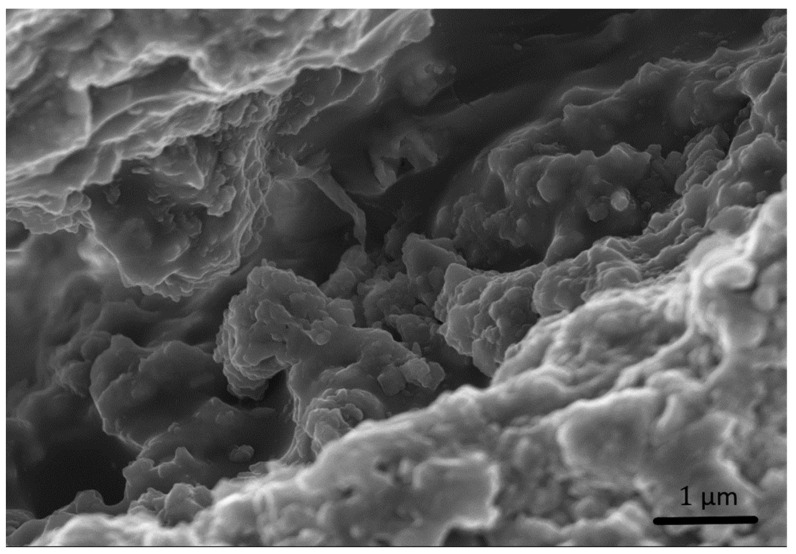
Secondary electron (SE) SEM image of a possible filament of cellulose detected in a crack and immersed in the forming gel.

SEM images confirm that a content of 0.3% fibrillated cellulose increases the compactness of the mortar, by reducing the presence of voids. Additionally, the aggregate to binder interphase, the interfacial transition zone (ITZ), appears to be also improved (Figure 15). The ITZ is considered the weakest element of mortars and concretes, as the first cracks leading to material failure generally originate there. Therefore, a better interaction between binder and aggregate can lead to an enhancement of mechanical properties. Moreover, the general reduction of cracks and voids may improve the durability of the material, as cracks can be the main point of entry for water or aggressive chemical ions [35]. The FC effect in strengthening the ITZ and reducing the crack formation has already been observed in Portland cement concrete [12]. For the materials described in this work these aspects may as well be connected to the tendency that the filaments have to form a 3D network (Figure 5). This effect was also described elsewhere upon application of similar cellulose materials [11]. In this proposed reinforcement mechanism, the key to have a significant effect of the fibres is the formation of a continuous network that connects the particles in the mortar. This mechanism would also explain why lower loads of fibres do not significantly improve the mechanical strength of the resulting materials. On the other hand, no clear evidence of a bridging effect by the FC was found. It must be noted, though, that given the fibre morphology and size, it is extremely complicated to confidently distinguish them from the hydraulic compounds formed during lime hydration (Figure 16).

**Figure 17:**
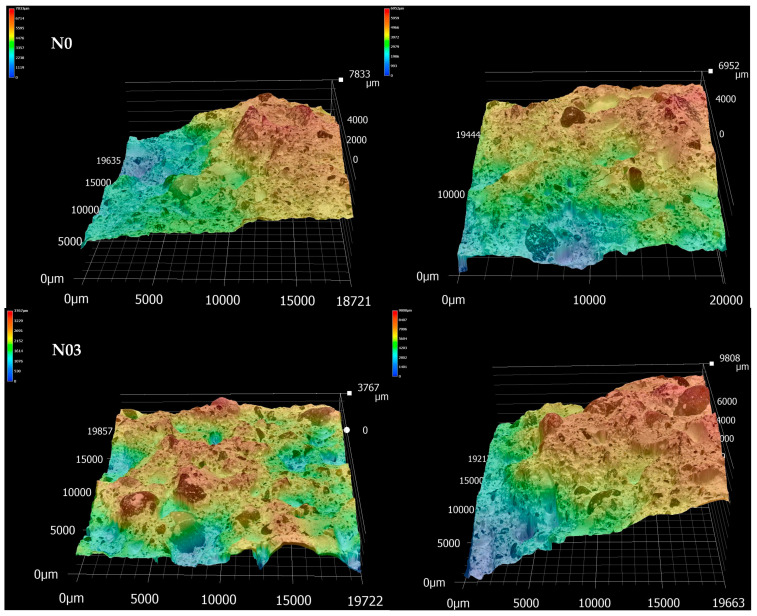
Representative 3D topographic images (20 mm × 20 mm ca) of the mortars fractured surfaces with 0% FC (N0) and 0.3% FC (N03).

The 3D topographies of the fractured surfaces of four representative samples are shown in Figure 17. The roughness parameters describing the two series are similar (Table 7). The calculated skewness shows that the peaks and valleys are normally distributed as the values are close to 0. The Sku for N0 defines that the distribution has a more collapsed shape. Overall, though, the 3D topographic images and the surface roughness parameters calculated did not underline any remarkable difference among the two series.

## 4. Conclusions

Fibrillated cellulose (FC) was incorporated in lime-based pastes and mortars from 0% up to 0.3% content by weight of binder (*w*/*w*).

The fibres do not affect the hydration of the mortars appreciably, as the thermal studies on the lime pastes underlined that the levels of hydraulic compounds formed are similar in all the samples. Nitrogen gas sorption analyses showed that 0.3% FC content determines a decrease in the pores volume in the range of 20–70 nm.

The different quantities of superplasticiser used for casting the mortars do not influence the setting and the early hydration in a significant way. The mechanical performance of mortars with 0.3% FC (N03) was indeed positively affected: the flexural and compressive *σ*_0_ were enhanced respectively by 57% and 44%. On the other hand, the study of the three-point bending test curves did not show any significant FC effect on the post-crack behaviour and deflection of the composite. The flexural elastic modulus E, on the contrary, was enhanced by 54% indicating the material behaves in a more rigid way. The FC effect on the mechanical properties can be connected to the microstructural changes that the fibres can induce in composites and that were already observed in Portland-cement-based concrete. These changes can be due to the higher elastic modulus of the crystalline portion of cellulose nanomaterials and their high specific surface area. Additionally, the interaction of the surface –OH groups with *CSH* and portlandite led to a stronger fibre–matrix bonding, which involves a better stress transfer from the matrix to the nanofibers, but in return can also cause an excessive embrittlement of the composite. The optical microscopy observations and the 3D topographies confirmed that FC does not affect the post-cracking behaviour of the mortars, while the scanning electron microscope (SEM) images showed that FC may promote a better adhesion between the binder and the aggregate strengthening the interfacial transition zone and therefore helping to get a more compact and homogeneous composite. Furthermore, the reinforcement mechanism may as well be connected to the continuous 3D network that the fibres form when incorporated in the fresh mortar.

In conclusion, FC is a good option for strengthening lime-based mortars to be used in green-buildings. Related composites are also of high interest for application in the field of historical building conservation, as natural hydraulic lime is compatible with aged and porous materials and improving the flexural strength eventually reduces the need for maintenance interventions. On the other hand, with the type of fibres used in the current study, it was not possible to act against crack propagation, delaying it with bridging mechanisms. Different kinds of cellulose fibres may be evaluated for this scope. Moreover, surface modification of fibres may be tested to improve their performance in the composite.

## Figures and Tables

**Table 1:** Sample codes of the lime pastes prepared with a water to binder ratio of 0.81 and relative weight content of fibrillated cellulose (FC) per weight of binder.

Mix	FC (%)
n0	0
n01	0.1
n02	0.2
n03	0.3

**Table 2:** Sample codes of the lime mortars prepared with w/b = 0.61 and relative fibrillated cellulose and superplasticiser (SP) dosage (*w*/*w*) per weight of binder.

Mix	FC (%)	SP (%)
N0	0	0.8
N01	0.1	1.0
N02	0.2	1.1
N03	0.3	1.2

**Table 3:** Flexural strength (f_fl_) of samples with 0% FC (N0) and 0.3% FC (N03).

Mix	Day 28	Day 45
N0	1.7 ± 0.4	2.2 ± 0.3
N03	2.5 ± 0.5	3.3 ± 0.3

**Table 4:** Compression strength (f_c_) of samples with 0% FC (N0) and 0.3% FC (N03).

Mix	Day 28	Day 45
N0	4.1 ± 0.3	4.8 ± 0.3
N03	5.8 ± 0.4	6.8 ± 0.5

**Table 5:** Results of three-point bending test stress–strain curves: total area (A) of the stress (MPa)-strain (%) relation, area (A_b_) after the break point (ε_b_) and flexural modulus (E) of mortars with 0% FC (N0) and 0.3% FC (N03).

Mix	A (MPa%)	A_b_ (MPa%)	ε_b_ (%)	E (MPa)
N0	0.6 ± 0.1	0.22 ± 0.07	0.48 ± 0.04	641 ± 113
N03	0.8 ± 0.1	0.31 ± 0.07	0.42 ± 0.04	986 ± 104

**Table 6:** Average experimental strength values and parameters obtained after Weibull fitting.

Mix	f_fl_ (MPa)	Flexural *σ*_0_ (MPa)	m	R^2^
**N0**	2.2	2.3	4.1	0.93
**N03**	3.3	3.6	7.0	0.94
	**f_c_ (MPa)**	**Compressive *σ*_0_ (MPa)**	**m**	**R^2^**
**N0**	4.8	5.2	6.3	0.97
**N03**	6.8	7.5	6.1	0.98

**Table 7:** Average surface roughness values obtained from 3D scans of fractured surfaces after three-point-bending tests.

Mix	*Sa* (µm)	*Sz* (µm)	*Sq* (µm)	*Ssk* (µm)	*Sku* (µm)
N0	1110 ± 322	6450 ± 1445	1341 ± 381	0.1 ± 0.2	2.4 ± 0.3
N03	1157 ± 352	6472 ± 1219	1324 ± 405	0.1 ± 0.4	2.7 ± 0.5

## Data Availability

The data presented in this study are available on request from the corresponding author.
